# Neighborhood Disadvantage and the Association of Hurricanes Sandy and Harvey With Veterans’ Mental Health

**DOI:** 10.1001/jamanetworkopen.2024.55013

**Published:** 2025-01-17

**Authors:** Caryn S. Yip, Peter J. Kaboli, Michael P. Jones, Margaret Carrel, Peter S. Thorne

**Affiliations:** 1 Department of Occupational and Environmental Health, University of Iowa, Iowa City; 2 Department of Internal Medicine, University of Iowa Carver College of Medicine, Iowa City; 3 Center for Comprehensive Access and Delivery Research and Evaluation, Iowa City Veterans Affairs Healthcare System, Iowa City; 4 Department of Biostatistics, University of Iowa, Iowa City; 5 Department of Geographical and Sustainability Sciences, University of Iowa, Iowa City

## Abstract

**Importance:**

Hurricanes are associated with a wide range of adverse health effects in the general population and are increasing in frequency and severity due to global climate change. Due to prior military exposures and distinct sociodemographic characteristics, US veterans may be more vulnerable than the general population to negative health effects of hurricanes.

**Objective:**

To evaluate whether acute care mental health visits among US veterans were associated with exposure to hurricanes.

**Design, Setting, and Participants:**

This retrospective cohort study consisted of US veterans enrolled in Veteran Health Administration (VHA) primary care and whose home addresses were in regions that were affected by Hurricanes Sandy or Harvey. The Hurricane Sandy cohort included data from 960 394 veterans between October 29, 2011, and October 28, 2016, and the Hurricane Harvey cohort included data from 795 746 veterans between August 25, 2016, and August 24, 2021. Affected regions were determined from Federal Emergency Management Agency designations; patient-level clinical data were obtained through VHA electronic health records. Statistical analysis was conducted from October 2022 to November 2023.

**Main Outcomes and Measures:**

The outcome of interest was acute care mental health visits, which included urgent care or emergency department visits or having an inpatient admission. The association between the outcome and exposure to hurricanes was estimated using Cox proportional hazards regression models with adjustments for multiple demographic and neighborhood characteristics, including neighborhood disadvantage, assessed using the Area Deprivation Index, and prior health status, assessed using the Care Assessments Need (CAN) score.

**Results:**

Of the 960 394 veterans in the Hurricane Sandy cohort, the mean (SD) age was 63 (16) years, and 895 726 (93.3%) were men; of the 795 746 veterans in the Hurricane Harvey cohort, the mean (SD) age was 59 (16) years, and 715 460 (89.9%) were men. For both hurricane cohorts, increasing neighborhood disadvantage was associated with an increased hazard of having a subsequent acute care mental health visit; this association was more pronounced among healthier veterans (Sandy cohort: 1-year hazard ratio [HR], 1.62 [95% CI, 1.53-1.71]; Harvey cohort: 1-year HR, 1.64 [95% CI, 1.54-1.74]) compared with veterans who had severe illness (Sandy cohort: HR, 1.22 [95% CI, 1.17-1.26]; Harvey cohort: HR, 1.21 [95% CI, 1.13-1.30]). After adjusting for baseline data, the association between hurricane exposure and our outcomes of interest was largely null. For the Hurricane Sandy cohort, those who were flooded and received individual assistance demonstrated a 1-year HR of 1.31 (95% CI, 0.81-2.12) if their CAN score was 80 or above and 0.86 (95% CI, 0.60-1.22) for a CAN score below 80. Similarly, those affected by Hurricane Harvey who were flooded and received individual assistance had a 1-year HR of 1.06 (95% CI, 0.99-1.14) for a CAN score of 80 or above and a 1-year HR of 0.98 (95% CI, 0.91-1.05) for a CAN score less than 80.

**Conclusions and Relevance:**

This study used a novel and highly granular approach to examine the associations of hurricanes with mental health outcomes. These findings are the first to show that hurricane exposure was not associated with adverse mental health outcomes among US veterans and illustrate the importance of considering preexisting regional differences when assessing the associations of hurricanes with mental health outcomes. The results suggest that neighborhood characteristics, rather than exposure to a hurricane, are the dominant determinants of mental health outcomes.

## Key Points

**Question:**

What are the associations between hurricane exposure and acute mental health visits among US veterans?

**Findings:**

In this cohort study that included 1 756 140 US veterans, increasing neighborhood disadvantage was associated with an increased hazard of having a subsequent acute care mental health visit. However, exposure to Hurricanes Sandy and Harvey were not associated with increased acute care mental health visits up to 4 years after the hurricanes.

**Meaning:**

This study illustrates the importance of considering preexisting regional differences before assessing the association of climate-exacerbated disasters with mental health outcomes between these regions.

## Teaser

This cohort study evaluates whether acute care mental health outcomes among US veterans were associated with exposure to hurricanes and whether demographic and neighborhood characteristics played a role in this association.

## Introduction

Hurricanes and tropical storms have been increasing in frequency and severity due to global climate change.^1^ Consequently, individuals living in hurricane-prone areas are increasingly exposed to environmental hazards, as well as social stressors.^2,3,4,5,6^ Hurricanes are associated with a wide range of adverse health conditions, including psychosocial effects. This is especially true for those displaced to temporary shelters, as this displacement results in separation from social support networks and creates a disruption in daily life.^7^

Numerous risk factors play a role in determining the degree to which people are affected by hurricanes.^8^ These can include factors related to the hurricane itself, such as severity and duration of flooding, extent of property damage, or displacement. There are also individualized factors that can affect the psychological distress experienced, such as prior experience with hurricanes, preexisting medical conditions, social support system, or disaster preparedness. These experiences can lead to mental health issues, such as depression, posttraumatic stress disorder (PTSD), or substance abuse.^7,9^ Our study evaluates the associations of Hurricanes Sandy and Harvey with mental health outcomes among US veterans.

Hurricane Sandy made landfall near Atlantic City, New Jersey, on October 29, 2012, causing extensive damage across several northeastern states. Major population centers in New Jersey and New York were greatly affected, as the storm caused widespread interruption to critical water and electrical services. Hurricane Sandy caused 159 deaths and cost an estimated $88.5 billion (after adjusting for the Consumer Price Index).^1^

Hurricane Harvey made landfall near Rockport, Texas, on August 25, 2017, as a category 4 hurricane, resulting in $160 billion (adjusted for the Consumer Price Index) in damages, at least 68 direct deaths, displacement of an estimated 40 000 people, and damage to more than 200 000 homes and businesses. Hurricane Harvey produced sustained heavy rainfall from August 24 through 31, with some affected areas receiving an excess of 127 cm.

Due to prior military exposures and distinct sociodemographic characteristics, such as advancing age^10,11^ and higher rates of current or past smoking,^12,13^ veterans may be more vulnerable than the general population to adverse health effects of hurricanes.^14,15^ In addition, the prevalence of serious mental illness is higher among veterans when compared with the overall adult population and among men, after adjustment for age, sex, and race and ethnicity.^16^ According to a 2015 study of primary care patients in the Veteran Health Administration (VHA), approximately 25% reported 1 or more mental illnesses.^14^

Prior research that assessed health outcomes associated with hurricanes relied on questionnaires and self-reported outcomes, used convenience sampling and insurance claims data, or focused on health care facilities rather than home residences.^17,18,19^ In contrast, our study used finer-scale data to assess hurricane exposure and patient-level clinical data that captured patient identifiers, health history, demographics, mental health visit dates, and standardized coding for diagnoses, medications, and procedures. The objective of this study was to evaluate the association between hurricane exposure and acute care mental health visits among US veterans. To our knowledge, this is the first study to evaluate the association of Hurricanes Sandy and Harvey with mental health outcomes in this population.

## Methods

### Study Design and Sample Selection

This study is a retrospective cohort study comprising participants drawn from the VHA, the largest integrated health care system in the US, providing care at 1255 health care facilities and serving 9 million enrolled veterans each year.^20,21^ Veterans were included if they were enrolled in VHA primary care, had complete demographic data, and had a geocoded address on file during the quarter the hurricane occurred. The Hurricane Sandy cohort included data from 960 394 veterans between October 29, 2011, and October 28, 2016, and the Hurricane Harvey cohort included data from 795 746 veterans between August 25, 2016, and August 24, 2021. This cohort study was approved by the institutional review board at the University of Iowa with a waiver of informed consent. Consent was waived because the study is a retrospective, secondary data analysis of health records with no direct contact with patients, in accordance with 45 CFR §46. This study followed the Strengthening the Reporting of Observational Studies in Epidemiology (STROBE) reporting guideline.

### Data Sources

We used data from (1) Housing Damage Maps from the US Department of Housing and Urban Development, (2) Disaster Declaration maps by the Federal Emergency Management Agency (FEMA), and (3) VHA electronic health records (EHRs). FEMA Housing Damage maps and Declared Disasters maps were used to categorize hurricane exposure.^22,23^ Data for Housing Damage maps were derived from home inspections and quantify personal property loss and damage resulting from the disasters. Declared Disasters maps categorize counties based on the types of funding received, which depends on their needs after the disaster. These datasets are publicly available as geographic information system shapefiles and geodatabases.

Patient-level clinical and demographic data were obtained from the VHA EHR included in the Corporate Data Warehouse. The Corporate Data Warehouse provides an integrated inpatient and outpatient EHR for patients at all VHA health care facilities and captures patient identifiers (eg, Global Positioning System coordinates for home, date of birth), demographics (eg, sex, age, self-reported race and ethnicity), health history, medication use, diagnostic test results, and other clinical details. The racial and ethnic categories were the US Census categories. Race and ethnicity were assessed because prior literature has indicated racial and ethnic variations in hurricane exposure and associated health outcomes.^24,25,26^

### Covariates

Microsoft SQL Server Management Studio, version 18.0 (Microsoft Corp) was used to retrieve patient data for veterans who lived in the study areas during each respective hurricane. ArcMap, version 10.5 (ESRI) and RStudio, version 4.1.2 (R Project for Statistical Computing) were used to overlay and link VHA patient data with Housing Damage Estimates by Block Group^22^ and FEMA Disaster Declaration^23^ shapefiles. Patient data were clipped to Veterans Integrated Service Network maps and then clipped to Housing Damage maps and FEMA disaster shapefiles in ArcMap, version 10.5 (ESRI). The sp package in R was used to link our point data with Area Deprivation Index (ADI) data from the University of Wisconsin’s Neighborhood Atlas.^27,28,29^

Hurricane exposure was categorized into 4 categories: no exposure, public assistance, individual assistance, and flooded with individual assistance (eAppendix in Supplement 1). Our study areas and exposure categories are shown in eFigure 1A in Supplement 1.

The *flooded with individual assistance* category was assigned under the assumption that veterans living in areas that were directly damaged by the hurricane would be most affected, due to factors such as loss of basic services, property loss and damage, and direct exposure to toxic agents.^30,31^ The *individual assistance* and *public assistance* categories were chosen under the assumption that those living in counties that received individual assistance would be more affected than those living in counties that received only public assistance.^32^

Additional variables in our models included prior health status and neighborhood disadvantage. Prior health status was included in our analysis through the inclusion of the Care Assessments Need (CAN) score, a analytic tool the VHA developed that estimates the likelihood of hospitalization or death within the next year for an individual patient compared with other patients.^33^ The score includes information on patient demographics, medical conditions, medication use, diagnoses, hospital visits, and health care utilization (eTable in Supplement 1). The CAN score is calculated each week and expressed as a percentile, ranging from 0 (lowest risk) to 99 (highest risk). Neighborhood disadvantage was included as a covariate through the ADI, a percentile index that ranks neighborhoods by socioeconomic disadvantage (eFigure 1B in Supplement 1). It includes factors for income, education, employment, and housing quality.^29,34,35^ In our analysis, ADI scores were recoded as 25% intervals and assigned a value between 1 (least disadvantaged) and 4 (most disadvantaged neighborhoods). Race and ethnicity were not included in our main analysis due to discrepancies in the available data, but a sensitivity analysis is available in eFigures 2 and 3 in Supplement 1.

### Outcomes

Our study examined the association between hurricane exposure and acute mental health visits using the following codes: depression (*International Classification of Diseases, Ninth Revision* [*ICD-9*], codes 296.2-296.3, 296.82, and 311; *International Statistical Classification of Diseases and Related Health Problems, Tenth Revision* [*ICD-10*], codes F32-F33), PTSD (*ICD-9* code 309.81, *ICD-10* code F43.1), and substance abuse disorder (*ICD-9* codes 303-305, *ICD-10* codes F10-F19). In our main analysis, we examined a combination of generalized anxiety disorder (*ICD-9* code 300.02, *ICD-10* code F41) and all aforementioned outcomes, referred to as *all mental health outcomes*. Acute care mental health visits, which included inpatient hospital admissions and urgent care or emergency department visits in which the visit’s primary diagnosis was an outcome of interest, were considered. Our outcome variable was time to first adverse event, regardless of category.

### Statistical Analysis

Statistical analysis was conducted from October 2022 to November 2023. The cohort study used Cox proportional hazards regression models to investigate the association between hurricane exposure and acute care mental health visits among US veterans. Data from 1 year before the hurricane to 4 years after the hurricane were analyzed. Models were adjusted for ADI and hurricane exposure. Time to first event was evaluated, where event was defined as the first acute care mental health visit for the conditions of interest. As is often done in the literature, suppose the time of the hurricane is taken as time 0 (ie, only adverse events after the hurricane are considered) and that the hazard ratio (HR) for each hurricane exposure region is estimated relative to the no-exposure (reference) region. However, a serious issue of potential bias arose. Initial Cox proportional hazards regression models using only the year prior to the hurricane (Table 1) showed preexisting differences in mental health outcomes among the regions that would later be affected by the hurricane.

**Table 1.** Prehurricane and Posthurricane Analysis for All Mental Health Outcomes^a^

Exposure	All mental health outcomes, HR (95% CI)			
	Hurricane Sandy		Hurricane Harvey	
	1 y Before (October 29, 2011, to October 28, 2012)	1 y After (October 29, 2012, to October 28, 2013)	1 y Before (August 25, 2016, to August 24, 2017)	1 y After (August 25, 2017, to August 24, 2018)
**CAN score <80**				
ADI quartile 1	1 [Reference]	1 [Reference]	1 [Reference]	1 [Reference]
ADI quartile 2	1.16 (1.06-1.25)	1.21 (1.14-1.28)	1.24 (1.13-1.37)	1.09 (1.00-1.20)
ADI quartile 3	1.31 (1.21-1.43)	1.36 (1.28-1.45)	1.47 (1.34-1.62)	1.29 (1.18-1.41)
ADI quartile 4	1.42 (1.29-1.55)	1.68 (1.57-1.79)	1.71 (1.56-1.88)	1.51 (1.39-1.65)
No exposure	1 [Reference]	1 [Reference]	1 [Reference]	1 [Reference]
Public assistance	1.15 (1.07-1.23)	1.19 (1.13-1.25)	1.13 (1.07-1.20)	0.99 (0.93-1.05)
Individual assistance	0.92 (0.77-1.09)	0.95 (0.84-1.07)	0.83 (0.70-0.98)	1.06 (0.91-1.22)
Flooded with individual assistance	0.82 (0.55-1.22)	1.10 (0.85-1.42)	1.24 (1.18-1.31)	1.29 (1.23-1.36)
**CAN score ≥80**				
ADI quartile 1	1 [Reference]	1 [Reference]	1 [Reference]	1 [Reference]
ADI quartile 2	1.09 (1.04-1.13)	1.08 (1.02-1.13)	1.10 (0.99-1.21)	1.08 (0.98-1.19)
ADI quartile 3	1.06 (1.02-1.11)	1.06 (1.00-1.11)	1.13 (1.03-1.25)	1.09 (0.99-1.19)
ADI quartile 4	1.20 (1.15-1.26)	1.15 (1.09-1.21)	1.20 (1.09-1.32)	1.22 (1.11-1.34)
No exposure	1 [Reference]	1 [Reference]	1 [Reference]	1 [Reference]
Public assistance	0.97 (0.94-1.01)	1.01 (0.97-1.05)	1.20 (1.14-1.26)	1.04 (0.98-1.09)
Individual assistance	1.18 (1.08-1.29)	1.14 (1.03-1.26)	0.81 (0.69-0.94)	0.98 (0.86-1.13)
Flooded with individual assistance	1.14 (0.96-1.37)	0.99 (0.79-1.23)	1.24 (1.18-1.30)	1.25 (1.19-1.30)

Abbreviations: ADI, Area Deprivation Index; CAN, Care Assessments Need; HR, hazard ratio.

^a^ The exposure categories 1 year prior to the hurricane match the future exposure zone exposure categories. The results of 8 separate Cox proportional hazards regression models (subset by CAN and divided by time period) are shown. Each row corresponds to a different parameter within the Cox proportional hazards regression model. The 95% CIs are the Wald confidence intervals for exponentiated coefficients. For example, the bottom row for Hurricane Harvey indicates that veterans living in the area that will be flooded 1 year hence already had an elevated HR (1.24) for all mental health outcomes compared with the no-exposure control group.

To address this issue, the analysis plan was now to estimate the change in HRs of adverse events among the 4 hurricane exposure regions, labeled *future exposure zones* (FEZs), that occur after the hurricane relative to what they were prior to the hurricane. Time 0 was now the time of the hurricane minus 1 year.

The Cox proportional hazards regression model for the analysis of time to adverse health event based on 1 year before and 1 year after the hurricane is as follows: λ_0_(*t*) exp [α′ADI + {β_1_ + γ_1_*H*(*t*)}*Z*_1_ + {β_2_ + γ_2_*H*(*t*)}*Z*_2_ + {β_3_ + γ_3_*H*(*t*)}*Z*_3_], where λ_0_(*t*) is the baseline hazard function and α′ = (α_1_, α_2_, α_3_) are the coefficients for the (second, third, fourth) quartiles of ADI. The time-dependent covariate *H*(*t*) = 0 prior to the hurricane and 1 after the hurricane. The non–time-dependent covariates (*Z*_1_, *Z*_2_, *Z*_3_) are indicators for the zones affected by the hurricane and labeled as *public assistance*, *individual assistance*, and *flooded with individual assistance*. These indicators allowed us to look for preexisting differences in areas that would later be affected by the hurricane (for further clarification of model interpretation, see eAppendix in Supplement 1).

This analysis plan addresses preexisting biases in the cohort and handles the effect of seasonality. As only time to the first outcome event is considered, participants are no longer included in the analysis after an event. Two separate models, subset by CAN score (<80, ≥80)^36^ and adjusted for ADI, FEZ, and hurricane exposure, were conducted. The HRs during various time points (6 weeks, 6 months, 1 year, and 4 years) after the hurricane were compared with HRs during the analogous period the previous year. Statistical analysis was completed in SAS statistical software, version 8.3 (SAS Institute Inc) using the PHREG procedure. Sensitivity analyses and separate analyses for depression, PTSD, and substance abuse can be found in the eAppendix in Supplement 1.

## Results

There were 1 528 325 and 1 046 367 veterans receiving VHA care living in our study area with an address on file during Hurricane Sandy and Hurricane Harvey, respectively (eFigure 1 in Supplement 1). After excluding veterans with incomplete covariate data, the final analytic cohort included 960 394 veterans (mean [SD] age, 63 [16] years; 895 726 [93.3%] men and 64 490 women [6.7%]; 10 078 American Indian or Alaska Native, Native Hawaiian or Pacific Islander, or Asian individuals [1.2%], 171 569 Black or African American individuals [19.6%], 12 926 Hispanic or Latino individuals [1.5%], and 679 798 White individuals [77.7%]) for Hurricane Sandy and 795 746 veterans (mean [SD] age, 59 [16] years; 715 460 [89.9%] men and 80 286 women [10.1%]; 11 274 American Indian or Alaska Native, Native Hawaiian or Pacific Islander, or Asian individuals [2.0%], 149 457 Black or African American individuals [26.3%], 66 914 Hispanic or Latino individuals [11.8%], and 340 254 White individuals [59.9%]) for Hurricane Harvey (Table 2). For the Hurricane Sandy cohort, the mean (SD) ADI was 51 (25), and the mean (SD) CAN score was 47 (30); for the Hurricane Harvey cohort, the mean (SD) ADI was 62 (23), and the mean CAN score was 43 (29) (Table 3).

**Table 2.** Demographic Characteristics

Characteristic	Individuals No. (%)	
	Hurricane Sandy (n = 960 394)	Hurricane Harvey (n = 795 746)
Hurricane exposure		
No exposure	752 188 (78.3)	576 657 (72.5)
Public assistance	177 044 (18.4)	101 651 (12.8)
Individual assistance	25 940 (2.7)	11 817 (1.5)
Flooded with individual assistance	5222 (0.5)	105 621 (13.3)
Age, mean (SD), y	63 (16)	59 (16)
Care Assessment Needs Score (CAN)		
<80	822 987 (85.7)	691 954 (87.6)
≥80	137 207 (14.3)	98 434 (12.5)
CAN score, mean (SD)	47 (30)	43 (29)
Area Deprivation Index Score		
Quartile 1 (least disadvantaged)	168 180 (17.5)	48 460 (6.1)
Quartile 2	318 968 (33.2)	198 178 (24.9)
Quartile 3	282 626 (29.4)	273 585 (34.4)
Quartile 4	190 620 (19.9)	275 523 (34.6)
Area Deprivation Index Score, mean (SD)	51 (25)	62 (23)
Race and ethnicity^a^		
American Indian or Alaska Native, Native Hawaiian or Pacific Islander, Asian	10 078 (1.2)	11 274 (2.0)
Black or African American	171 569 (19.6)	149 457 (26.3)
Hispanic or Latino	12 926 (1.5)	66 914 (11.8)
White	679 798 (77.7)	340 254 (59.9)
Decline to answer or unknown	28 182 (3.0)	57 305 (6.9)
Sex		
Male	895 726 (93.3)	715 460 (89.9)
Female	64 490 (6.7)	80 286 (10.1)

^a^ Totals for race and ethnicity do not sum to the total number of individuals due to discrepancies in the data.

**Table 3.** Demographic Characteristics, Categorized by Exposure Level

Characteristic	CAN score, mean (SD)	ADI, mean (SD)	Age, mean (SD), y
Hurricane Sandy	47 (30)	51 (25)	63 (16)
No exposure	50 (29)	55 (24)	60 (16)
Public assistance	51 (30)	39 (23)	59 (16)
Individual assistance	48 (28)	37 (18)	60 (16)
Flooded with individual assistance	51 (27)	39 (24)	63 (16)
Hurricane Harvey	43 (29)	62 (23)	59 (16)
No exposure	45 (29)	65 (22)	61 (16)
Public assistance	42 (29)	57 (24)	57 (16)
Individual assistance	46 (28)	67 (19)	63 (16)
Flooded with individual assistance	45 (29)	59 (24)	59 (16)

Abbreviations: ADI, Area Deprivation Index; CAN, Care Assessments Need.

Our pre-post analysis showed that there were existing differences in HRs for all mental health visits before each hurricane (Table 1). Prior to Hurricane Sandy, regions that ultimately received public assistance or individual assistance were already positively associated with our outcome (1-year public assistance: HR, 1.15 [95% CI, 1.07-1.23] among those with a CAN score <80; 1-year individual assistance: HR, 1.18 [95% CI, 1.08-1.29] among those with a CAN score ≥80). Prior to Hurricane Harvey, areas that would later receive public assistance and areas that would be flooded with individual assistance were positively associated with our outcomes of interest in both strata (1-year public assistance among those with a CAN score <80: HR, 1.13 [95% CI, 1.07-1.20] and 1-year flooded with individual assistance among those with a CAN score <80: HR, 1.24 [95% CI, 1.18-1.31]; 1-year public assistance among those with a CAN score ≥80: HR, 1.20 [95% CI, 1.14-1.26], 1-year flooded with individual assistance among those with a CAN score ≥80: HR, 1.24 [95% CI, 1.18-1.30]). The analysis also showed similar HRs for each exposure region after both hurricanes.

**Figure 1.**
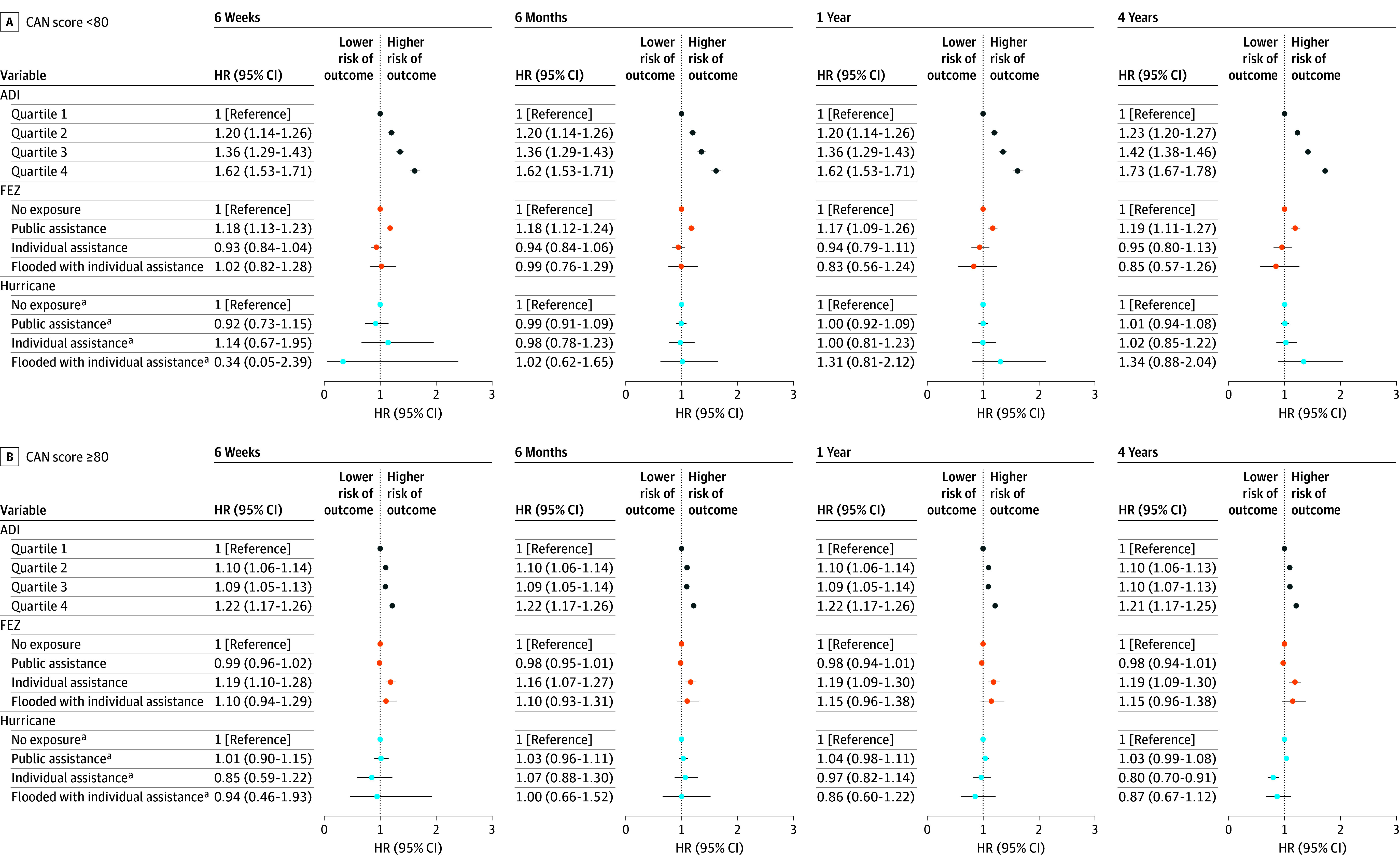
Hurricane Sandy, All Mental Health Outcomes Hazard ratios (HRs) and 95% CIs for the association of Area Deprivation Index (ADI) with exposure to Hurricane Sandy and with urgent care or emergency department visits and inpatient hospitalizations for all mental health outcomes (depression, generalized anxiety disorder, post-traumatic stress disorder, and substance abuse), within strata of Care Assessments Need (CAN) scores across all time periods. Area Deprivation Index is a measure of neighborhood disadvantage. Future exposure zone (FEZ) describes where each veteran resides but uses future hurricane exposure (Hurricane Sandy or Hurricane Harvey) to determine which exposure category they fall under. ^a^These values represent how much greater the posthurricane HR is compared with the prehurricane HR.

**Figure 2.**
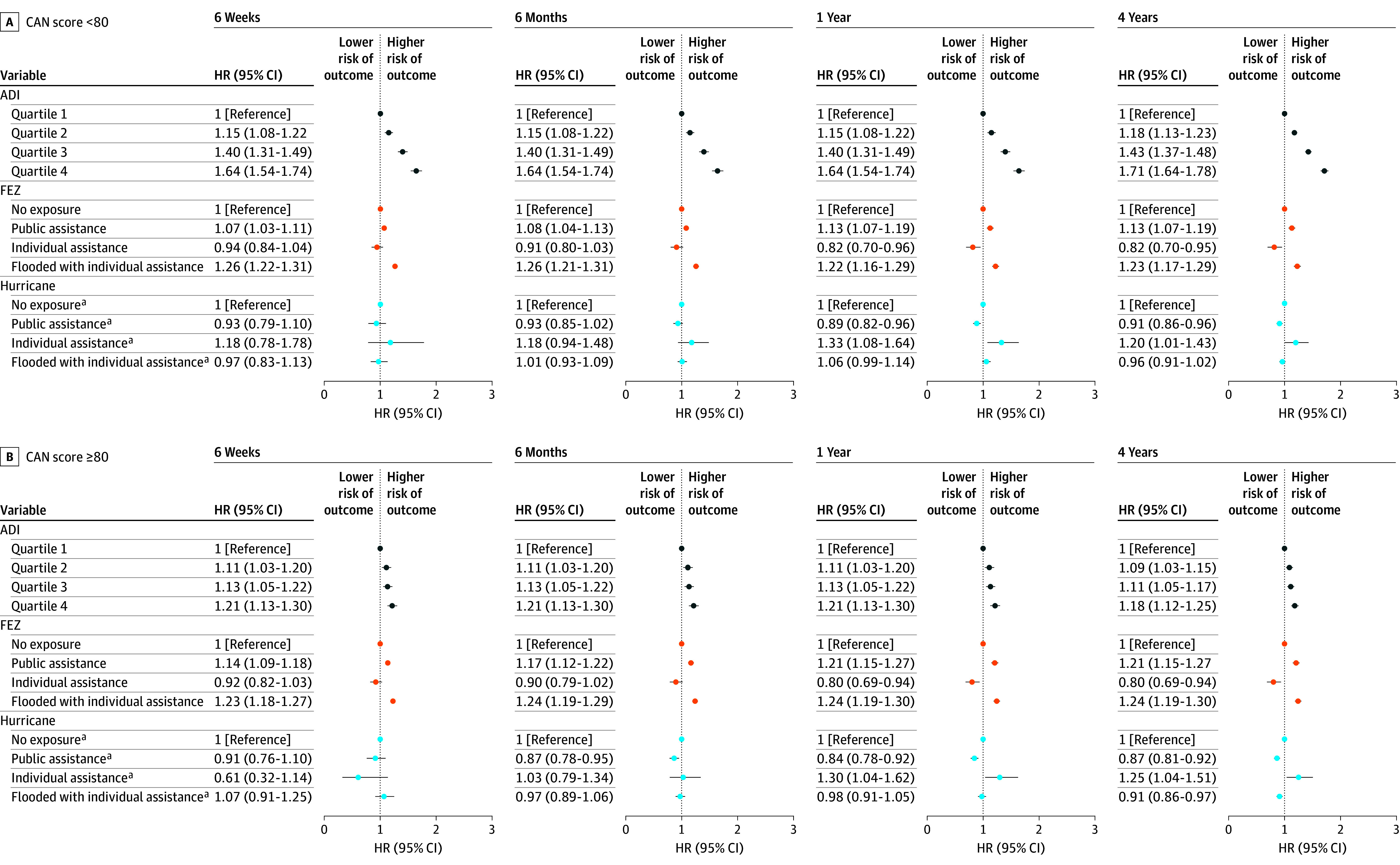
Hurricane Harvey, All Mental Health Outcomes Hazard ratios (HRs) and 95% CIs for the association of Area Deprivation Index (ADI) with exposure to Hurricane Harvey and with urgent care or emergency department visits and inpatient hospitalizations for all mental health outcomes (depression, generalized anxiety disorder, post-traumatic stress disorder, and substance abuse), within strata of Care Assessments Need (CAN) scores across all time periods. Area Deprivation Index is a measure of neighborhood disadvantage. Future exposure zone (FEZ) describes where each veteran resides but uses future hurricane exposure (Hurricane Sandy or Hurricane Harvey) to determine which exposure category they fall under. ^a^These values represent how much greater the posthurricane HR is compared with the prehurricane HR.

During the 4-year follow-up period, there were 96 881 outcome events for the Hurricane Sandy cohort and 99 298 outcome events for the Hurricane Harvey cohort. Across all health outcomes and time periods, models for both cohorts show a positive association between ADI and outcome events, particularly among veterans with a CAN score less than 80 (Figure 1 and Figure 2; eFigures 4-9 in Supplement 1). At the 1-year period for all mental health outcomes, veterans in the Hurricane Sandy cohort within ADI quartile 4 with a CAN score less than 80 had an HR of 1.62 (95% CI, 1.53-1.71), and those with a CAN score of 80 or more had an HR of 1.22 (95% CI, 1.17-1.26), compared with the least disadvantaged veterans (ADI quartile 1) in the same subset. In the 1-year post–Hurricane Harvey period, veterans within ADI quartile 4 with a CAN score less than 80 had an HR of 1.64 (95% CI, 1.54-1.74), and those with a CAN score of 80 or more had an HR of 1.21 (95% CI, 1.13-1.30). Subanalyses for the separate categories of mental health outcomes yielded similar results for ADI, with the exception of PTSD (eFigures 6 and 7 in Supplement 1).

There was a consistent but nonmonotonic association for FEZs across our models (Figure 1 and Figure 2), but the results showed that there were frequently preexisting differences between exposure groups, suggesting that some areas that would later be affected by hurricanes exhibited higher baseline levels of mental illnesses. There was a positive association between FEZ: flooded with individual assistance and all mental health outcomes in the Hurricane Harvey cohort in both strata (1 year: HR, 1.22 [95% CI, 1.16-1.29] among those with a CAN score <80; HR, 1.24 [95% CI, 1.19-1.30] among those with a CAN score ≥80). When accounting for FEZs, associations between hurricane exposure and health outcomes were largely null, although there were a few significant results among the Hurricane Harvey cohort. Considering all mental health outcomes in the public assistance group, the 1-year posthurricane HR for individuals with a CAN score less than 80 was 0.89 (95% CI, 0.82-0.96) times greater than (ie, protective) the prehurricane HR, and the 1-year posthurricane HR for individuals with a CAN score of 80 or more was 0.84 (95% CI, 0.78-0.92) times greater than the prehurricane HR. In the flooded-with-individual-assistance group, the 4-year posthurricane HR for individuals with a CAN score of 80 or more was 0.91 (95% CI, 0.86-0.97) times greater than the prehurricane HR.

## Discussion

To our knowledge, this is the first study to examine the association between hurricane exposure and adverse mental health outcomes, while accounting for preexisting differences, with this sample size and granularity. After adjusting for preexisting differences between exposure regions, we found that hurricane exposure was largely not significantly associated with acute care mental health visits among US veterans exposed to Hurricane Sandy or Hurricane Harvey. Instead, neighborhood disadvantage was strongly associated with adverse mental health outcomes, especially among healthier veterans.

The use of Cox proportional hazards regression models enabled us to include individual-level covariate data and time-varying covariates. The results from our pre-post analysis, which showed significant HRs in regions that would later be affected by the hurricanes, necessitate our inclusion of the FEZ covariate in our final models to account for baseline bias. We also ran a “negative control” model to check for residual bias from unmeasured confounding by examining gastrointestinal bleeding as our outcome of interest and found no significant association.

Our results support prior literature that indicates high levels of neighborhood disadvantage are associated with a greater likelihood of poor mental health outcomes.^37,38^ There was a strong positive association between ADI and acute care mental health visits after adjusting for hurricane exposure; this finding was consistent for both hurricanes and across all time periods. The association was attenuated among the veterans with severe illness, possibly because ADI does not matter as much when one’s health needs are already so high and health care is guaranteed.

Prior research that studied hurricane exposure in the general population reported that increased exposure to hurricanes was significantly associated with a greater likelihood of depression, PTSD, and anxiety.^39,40,41^ In contrast, our study reported no clear association between exposure to Hurricanes Sandy or Harvey during the 6-week, 6-month, 1-year, or 4-year periods after the hurricane after adjusting for neighborhood disadvantage and any baseline bias between exposure groups established through use of the FEZ, except in the public assistance group 1 year after Hurricane Harvey. One potential reason for this result is that the public assistance group had a lower ADI and CAN score than our reference group (Table 3). Another possibility is that this area has more experience with major hurricanes because Louisiana was affected by both Hurricanes Katrina and Rita. To our knowledge, this is the first study to track individuals before and after the storm, while adjusting for existing differences at baseline. Results from our pre-post analysis show that had we not included FEZ in our main models, we would have found a false-positive association between hurricane exposure and our outcomes, demonstrating the importance of including a variable that captures baseline data. This finding is novel.

Although veterans are generally in poorer health compared with nonveterans, and potentially more susceptible to hurricane-related stressors, the results from the present study are inconsistent with previous findings. Our study evaluated 4 different time points and found a persistent null effect for most of the exposure groups, indicating that the association of a hurricane with mental health outcomes appears to stay consistent over time. One phenomenon to consider is posttraumatic growth, defined as positive, meaningful psychological changes as a result of traumatic or stressful life events.^42,43^ In a nationally representative sample of US veterans, 1 study found that half of all veterans, and nearly 75% of those who screened positive for PTSD, reported at least moderate posttraumatic growth in association with their worst traumatic event.^42^ Veterans who experience hurricanes may discover an increased sense of purpose in rebuilding their communities, a greater appreciation of life, or deeper relationships with their social support network.

### Strengths and Limitations

Our study has some strengths. The biggest strength was our high level of granularity, as we were able to link individual-level exposure and covariate data with individual-level outcome data. We used a novel approach to adjust for any preexisting inequities in our study population to reduce baseline bias and exposure misclassification. Another key strength is the sample size, which provided sufficient power to evaluate our health outcomes at various time points. This study also relied on administrative data, which are not subject to self-reporting bias.

This study also has several limitations. First, the present study did not capture use of non-VHA services, even though some veterans may be dually covered by a private health care plan or Medicare. Our study attempted to address this limitation by including only participants enrolled in VHA primary care. Second, while our data were not subject to self-report bias, there may have been misdiagnoses or underdiagnoses of mental disorders. Third, there is potential for exposure misclassification if the veteran experienced flooding but lived in a census block group with fewer than 10 damaged homes as determined by FEMA. Fourth, the study may not be generalizable to the general population, as veterans are not representative of US demographics in terms of age or sex.

## Conclusions

As climate change continues to alter hurricane behavior, it is imperative that we learn from our past experiences to build resilience in our communities and our health care system. To our knowledge, this cohort study was the first to use individual-level exposure and outcome data and adjust for preexisting biases to evaluate whether hurricane exposure is associated with adverse mental health outcomes. After accounting for these differences, our study concluded that hurricane exposure was not significantly associated with adverse mental health outcomes among US veterans. However, increasing neighborhood disadvantage was associated with negative mental health outcomes. Our results suggest that neighborhood characteristics, rather than exposure to a hurricane, are the dominant determinants of mental health outcomes among veterans.

## Notes

Supplement 1. **eAppendix.** Detailed Methods
eReferences.
**eFigure 1.** Map of Study Area **eTable.** Care Assessment Needs Score Model Terms **eFigure 2.** Hurricane Sandy (Including Race/Ethnicity), All Mental Health Outcomes **eFigure 3.** Hurricane Harvey (Including Race/Ethnicity), All Mental Health Outcomes **eFigure 4.** Hurricane Sandy, Depression **eFigure 5.** Hurricane Harvey, Depression **eFigure 6.** Hurricane Sandy, Post-Traumatic Stress Disorder (PTSD) **eFigure 7.** Hurricane Harvey, Post-Traumatic Stress Disorder (PTSD) **eFigure 8.** Hurricane Sandy, Substance Abuse Disorder **eFigure 9.** Hurricane Harvey, Substance Abuse Disorder

Supplement 2.
Data Sharing Statement
