# Supplementary material for: Neighborhood Disadvantage and the Association of Hurricanes Sandy and Harvey With Veterans’ Mental Health
Source: JAMA Netw Open. 2025 Jan 17;8(1):e2455013. doi: 10.1001/jamanetworkopen.2024.55013 (PMC11742530; doi:10.1001/jamanetworkopen.2024.55013)
Supplement: Supplement 1. — eAppendix. Detailed Methods eReferences. eFigure 1. Map of Study Area eTable. Care Assessment Needs Score Model Terms eFigure 2. Hurricane Sandy (Including Race/Ethnicity), All Mental Health Outcomes eFigure 3. Hurricane Harvey (Including Race/Ethnicity), All Mental Health Outcomes eFigure 4. Hurricane Sandy, Depression eFigure 5. Hurricane Harvey, Depression eFigure 6. Hurricane Sandy, Post-Traumatic Stress Disorder (PTSD) eFigure 7. Hurricane Harvey, Post-Traumatic Stress Disorder (PTSD) eFigure 8. Hurricane Sandy, Substance Abuse Disorder eFigure 9. Hurricane Harvey, Substance Abuse Disorder [file jamanetwopen-e2455013-s001.pdf]

Supplementary Online Content

Yip CS, Kaboli PJ, Jones MP, Carrel M, Thorne PS. Neighborhood disadvantage and the association of Hurricanes Sandy and Harvey with veterans' mental health. *JAMA Netw Open.* 2025;8(1):e2455013. doi:10.1001/jamanetworkopen.2024.55013

eAppendix. Detailed Methods

eReferences.

eFigure 1. Map of Study Area

eTable. Care Assessment Needs Score Model Terms

eFigure 2. Hurricane Sandy (Including Race/Ethnicity), All Mental Health Outcomes

eFigure 3. Hurricane Harvey (Including Race/Ethnicity), All Mental Health Outcomes

eFigure 4. Hurricane Sandy, Depression

eFigure 5. Hurricane Harvey, Depression

eFigure 6. Hurricane Sandy, Post-Traumatic Stress Disorder (PTSD)

eFigure 7. Hurricane Harvey, Post-Traumatic Stress Disorder (PTSD)

eFigure 8. Hurricane Sandy, Substance Abuse Disorder

eFigure 9. Hurricane Harvey, Substance Abuse Disorder

This supplementary material has been provided by the authors to give readers additional information about their work.

© 2025 Yip CS et al. *JAMA Network Open.*

eAppendix. Detailed Methods

Study Design and Sample Selection

The study is a retrospective longitudinal cohort study where participants were drawn from VHA, America's largest integrated healthcare system, providing care at 1,255 healthcare facilities and serving 9 million enrolled veterans each year.[1,2] Veterans were included if they were enrolled in VHA primary care, under the assumption that continuity of care would result in more accurate diagnoses and documentation and less loss-to-follow-up. In addition, veterans needed complete demographic data and a geocoded address on file during the quarter the hurricane occurred, as an address was required to ascertain exposure status. This study was approved by the University of Iowa institutional review board (IRB-03 #202006520).

Covariates

Hurricane exposure was categorized into four categories: no exposure, public assistance, individual assistance, and flooded with individual assistance. Exposure categories are mutually exclusive and exhaustive. The 'flooded' category utilized the Housing Damage maps and captured veterans who lived in block groups most affected by the hurricane. 'Individual assistance' and 'public assistance' captured veterans who lived in counties that received some individual assistance or only public assistance from FEMA, respectively. The 'no exposure' category consisted of veterans who lived in the study area during the hurricane but were not included in the other groups. Individuals in this group were not subject to evacuation orders and experienced little damage. Our study areas and exposure categories are shown in Figure 1a.

The 'flooded with individual assistance' category was assigned under the assumption that veterans living in areas that were directly damaged by the hurricane would be most affected, due to factors such as loss of basic services, property loss and damage, and direct exposure to toxic

© 2025 Yip CS et al. *JAMA Network Open.*

agents.[3,4] The 'individual assistance' and 'public assistance' categories were chosen under the assumption that those living in counties that received individual assistance would be more affected than those living in counties that only received public assistance.[5] The only exceptions to this distinction were Tarrant and Dallas County, TX. While these two counties were not directly affected by the hurricane, they opened shelters for Hurricane Harvey evacuees and received public assistance from FEMA. Since hurricane exposure assessment was based on home addresses during the hurricane, Tarrant and Dallas County were classified as 'no exposure' to reduce exposure misclassification.

Statistical Analysis

The study utilized Cox regression models to investigate the association between hurricane exposure and acute care mental health visits in U.S. veterans. Data beginning October 29, 2011 through October 28, 2016 were examined for Hurricane Sandy and data beginning August 25, 2016 through August 25, 2021 were examined for Hurricane Harvey. Models were adjusted for ADI and hurricane exposure. Time-to-first-event was evaluated, where "event" was defined as the first acute care mental health visit for the condition(s) of interest. As is often done in the literature, suppose the time of the hurricane is taken as Time 0, i.e. only adverse events post-hurricane are considered, and that the hazard ratio for each hurricane exposure region is estimated relative to the no exposure (reference) region. However, a serious issue of potential bias arose. Initial Cox models using only the year prior to the hurricane (Table 2) showed pre-existing differences in mental health outcomes among the regions that would later be affected by the hurricane.

To address this issue, the analysis plan was now to estimate the change in hazard ratios of adverse events among the four hurricane exposure regions, labeled Future Exposure Zones (FEZ), that occur after the hurricane. Time 0 was now Time of Hurricane minus 1-year (H-1yr).

© 2025 Yip CS et al. *JAMA Network Open.*

The Cox regression model for the analysis of time to adverse health event based on 1-year prior and 1-year post-hurricane is:

$$\lambda_0(t)\ \exp\{\alpha' ADI + \beta_1 Z_1 + \beta_2 Z_2 + \beta_3 Z_3 + \gamma_1 Z_1 H(t) + \gamma_2 Z_2 H(t) + \gamma_3 Z_3 H(t)\}$$

where $\lambda 0(t)$ is the baseline hazard function and $\alpha' = (\alpha_1, \alpha_2, \alpha_3)$ are the coefficients for the (second, third, fourth) quartiles of ADI. The time-dependent covariate $H(t) = 0$ prior to the hurricane and = 1 after the hurricane. The non-time-dependent covariates (Z_1, Z_2, Z_3) are indicators for the zones affected by the hurricane and labeled as public assistance, individual assistance, and flooded with individual assistance. The reference group is no exposure. These will allow us to look for pre-existing differences in areas that will later be affected by the hurricane. This hazard model can be equivalently written as $\lambda_0(t)$ exp $[\alpha' ADI + \{\beta_1 + \gamma_1 H(t)\} Z_1 + \{\beta_2 + \gamma_2 H(t)\} Z_2 + \{\beta_3 + \gamma_3 H(t)\} Z_3]$. Adjusted for ADI, the pre-hurricane hazard ratio of zone j versus the no exposure zone is $\exp(\beta j)$ and the corresponding post-hurricane hazard ratio is $\exp(\beta j + \gamma j)$, for $j = 1, 2, 3$. Hence, the post-hurricane zone-j versus no exposure zone hazard ratio is $\exp\{\gamma j\}$ times that of the pre-hurricane hazard ratio. Values greater than 1 indicate a detrimental effect of the hurricane in zone j. The null hypothesis of no hurricane effect on zone j is a test of $\gamma j = 0$. The model for comparing 6 months post hurricane to the analogous 6-month period before the hurricane is similar but being sure just to only use events from the two 6-month periods.

This analysis plan addresses pre-existing biases in the cohort and handles the effect of seasonality. As only time-to-first-adverse-health-event is considered, subjects are no longer included in the analysis after an event. A counting process survival dataset was created following the methodology laid out by Andersen and Gill.[6] Follow-up time was counted in days. The model was stratified by CAN Score (< 80, ≥80)[7] and adjusted for ADI and hurricane exposure. Hazard rates during the 6-

© 2025 Yip CS et al. *JAMA Network Open.*

week, 6-month, and 1-year timepoint following the hurricane were compared to hazard rates during the analogous period the previous year. Statistical analysis was completed in SAS statistical software version 8.3 using the PHREG procedure.

Results

There were a total of 9,725,550 VHA enrollees alive during Hurricane Sandy and 8,471,115 alive during Hurricane Harvey. For those alive during Sandy, the average age was 58 and average CAN was 46. Those alive during Harvey were on average 60 years of age and had an average CAN of 46. 78% of those alive during Sandy and 93% of those alive during Harvey had a geocoded address.

There were 1,528,325 and 1,046,367 veterans receiving VHA care living in our study area with an address on file during Hurricane Sandy and Hurricane Harvey, respectively (Figure 1). After excluding veterans with incomplete covariate data, our final analytic cohort was 960,394 veterans (Sandy) and 795,746 (Harvey) (Table 1). For Hurricane Sandy, 93.27% were male, mean ADI was 51, mean CAN was 46, and mean age was 63. There were 475,623 veterans under the age of 65 and 484,771 65 years of age and older. The majority (89.91%) of our Harvey cohort were also male, and on average they were younger (mean age: 59), less sick (mean CAN: 43), and more disadvantaged (mean ADI: 62) compared to our Sandy cohort.

© 2025 Yip CS et al. *JAMA Network Open.*

eReferences.

1. Veterans Health Administration. Veterans Health Administration. Published April 3, 2020. Accessed April 13, 2020. https://www.va.gov/health/index.asp

2. Veterans Health Administration. Interactive US Map - Locations. Published October 12, 2018. Accessed April 13, 2020. https://www.va.gov/directory/guide/map.asp?dnum=1

3. Goldmann E, Galea S. Mental Health Consequences of Disasters. *Annu Rev Public Health*. 2014;35(1):169-183. doi:10.1146/annurev-publhealth-032013-182435

4. Mendell Mark J., Mirer Anna G., Cheung Kerry, Tong My, Douwes Jeroen. Respiratory and Allergic Health Effects of Dampness, Mold, and Dampness-Related Agents: A Review of the Epidemiologic Evidence. *Environ Health Perspect*. 2011;119(6):748-756. doi:10.1289/ehp.1002410

5. Federal Emergency Management Agency. Understanding Individual Assistance and Public Assistance. FEMA. Published July 20, 2015. Accessed April 29, 2020. https://www.fema.gov/news-release/2015/07/20/understanding-individual-assistance-and-public-assistance

6. Andersen PK, Gill RD. Cox’s Regression Model for Counting Processes: A Large Sample Study. *Ann Stat*. 1982;10(4):1100-1120.

7. Stephan Fihn, Tami Box. Care Assessment Need (CAN) Score and the Patient Care Assessment System (PCAS): Tools for Care Management. Presented at: June 27, 2013; Veterans Health Administration Office of Informatics and Analytics.

© 2025 Yip CS et al. *JAMA Network Open.*

eFigure 1. Map of Study Area

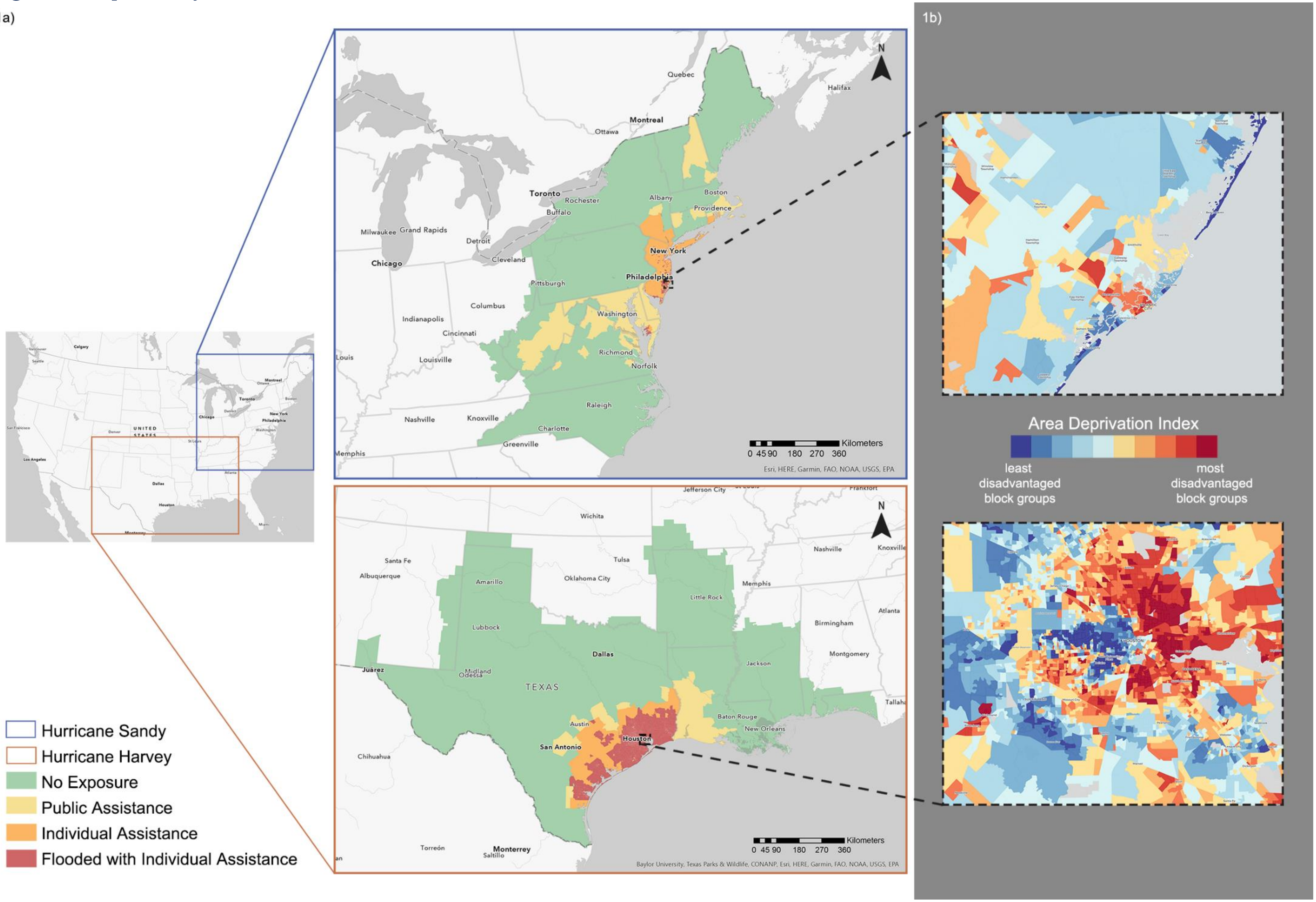

© 2025 Yip CS et al. *JAMA Network Open.*

eFigure 1a. Maps of our study area and exposure categories. The blue-bound box shows the study area for Hurricane Sandy while the orange-bound box shows the study area for Hurricane Harvey.

eFigure 1b. A close-up map showing the Area Deprivation Index of an area that was flooded after Hurricane Sandy and Hurricane Harvey. These maps show the large variability in deprivation between neighborhoods.

© 2025 Yip CS et al. *JAMA Network Open.*

eTable. Care Assessment Needs Score Model Terms

The Care Assessment Need score is a predictive analytic tool VHA developed that reflects the likelihood of hospitalization or death within the next year in an individual patient compared with other patients. CAN scores are automatically calculated each week using data from the VA's national electronic health record and include the following variables.

Demographics	Chronic Illness	Utilization	Vital Signs	Pharmacy	Interactions
• Age (≥ 65)<br>• Sex<br>• Marital status<br>• Service connection ≥ 50%	• Deyo-Charlson score<br>• Hierarchical Condition Categories<br>• Myocardial infarction/unstable angina/ coronary artery bypass grafting<br>• Respiratory failure<br>• Hypertension<br>• Stroke<br>• Renal failure<br>• Chronic obstructive pulmonary disease<br>• Atrial fibrillation<br>• Atherosclerotic Peripheral Vascular Disease<br>• Pneumonia<br>• Diabetes<br>• Liver disease<br>• Malnutrition<br>• Dementia<br>• Functional disease<br>• Metastatic Cancer<br>• Trauma<br>• Psych disease<br>• Post-traumatic stress disorder<br>• Depression	• Outpatient visits (> 4)<br>• Primary care visits (> 1)<br>• Cardiology visits<br>• Pulmonology visits<br>• Mental health visits<br>• ER visits (> 1 last year)<br>• Other visits (> 3)<br>• Recent admission<br>• BDOC (1-10 v. 0)<br>• Number of providers (>3)	• Systolic and diastolic blood pressure<br>• Heart rate (> 85)<br>• Respiratory rate (≥ 20)<br>• Body Mass Index (<25)	• Total number of refills (>0)<br>• Angiotensin-converting enzyme inhibitors and angiotensin II receptor blockers<br>• Alpha-blocker<br>• Nebulized drugs<br>• Antiplatelet drugs<br>• Anti-depressants<br>• Antipsychotics<br>• Benzodiazepine<br>• Beta-blockers<br>• Bumetanide or Torsemide<br>• Thiazides<br>• Calcium channel blockers<br>• Digoxin<br>• Furosemide<br>• Insulin<br>• Metolazone<br>• Metformin<br>• Nitrate-long-acting drugs<br>• Non-steroidal anti-inflammatory drugs<br>• Lipid lowering drugs<br>• P-par-gamma-agonists<br>• K+ sparing diuretic<br>• Oral steroids<br>• Warfarin	• 18 interaction terms

© 2025 Yip CS et al. *JAMA Network Open.*

eFigure 2. Hurricane Sandy (Including Race/Ethnicity), All Mental Health Outcomes

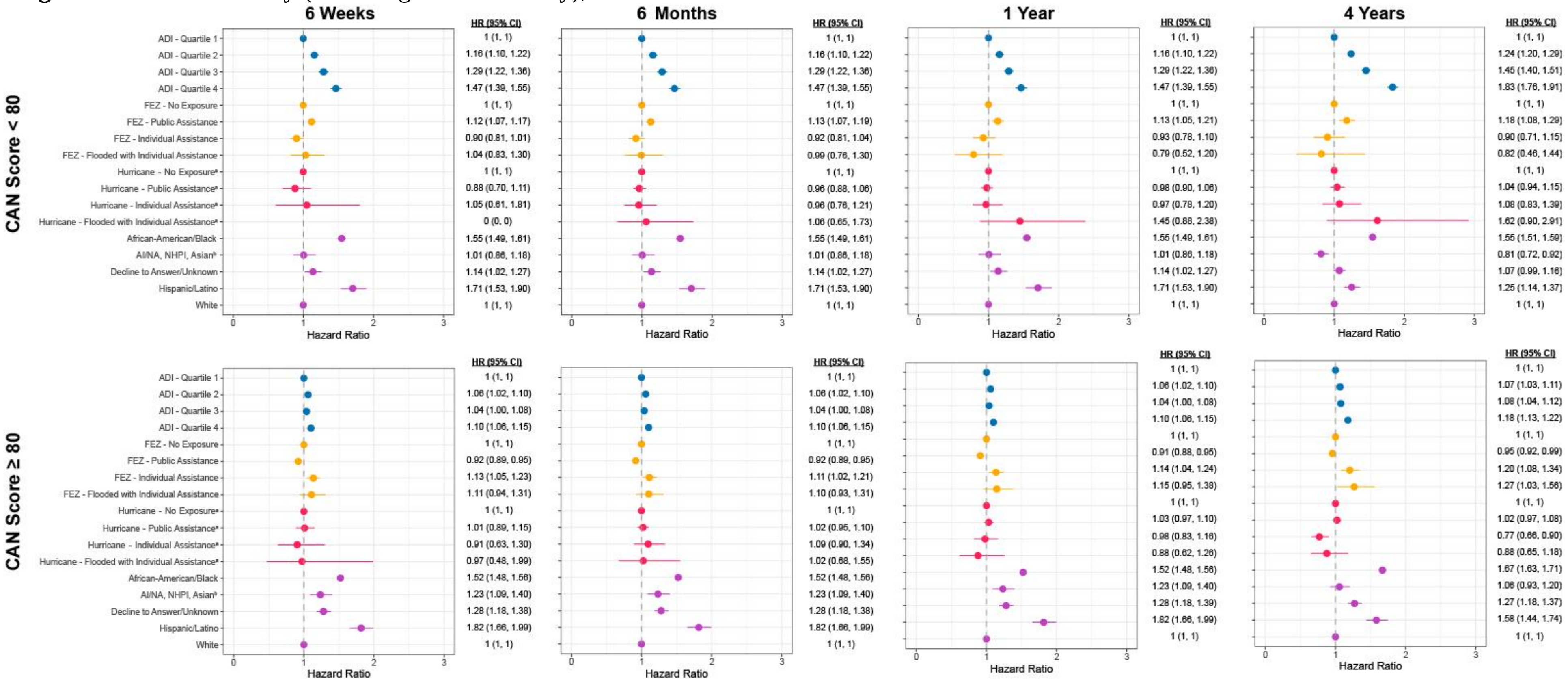

eFigure 2. Hazard ratio and 95% confidence intervals for the relationship between ADI, FEZ, and exposure to Hurricane Sandy, and UC/ED visits and in-patient hospitalizations for all mental health outcomes (depression, generalized anxiety disorder, PTSD, and substance abuse), subset by Care Assessment Needs (CAN) Scores and across all time periods. Area Deprivation Index (ADI) is a measure of neighborhood disadvantage. Future Exposure Zone (FEZ) describes where each veteran resides but uses future hurricane exposure to determine which exposure category they fall under. Race/ethnicity data was ascertained from Veterans Health Association electronic health records and are self-reported data.

[a]These values represent how much greater the post-hurricane hazard ratio is compared to the pre-hurricane hazard ratio.

[b]AI/NA = American-Indian/Native Alaskan, NHPI = Native Hawaiian and Other Pacific Islander

© 2025 Yip CS et al. *JAMA Network Open.*

eFigure 3. Hurricane Harvey (Including Race/Ethnicity), All Mental Health Outcomes

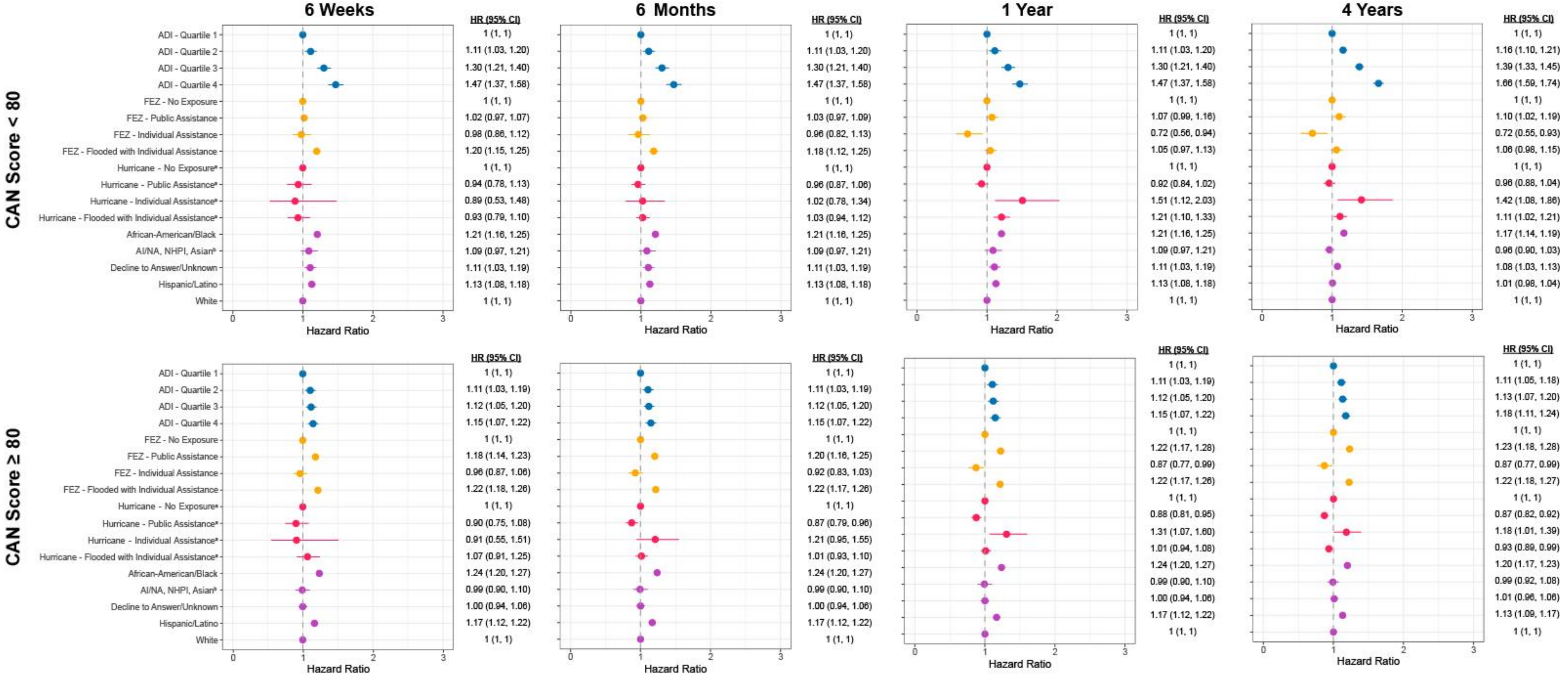

eFigure 3. Hazard ratio and 95% confidence intervals for the relationship between ADI, FEZ, and exposure to Hurricane Harvey, and UC/ED visits and in-patient hospitalizations for all mental health outcomes (depression, generalized anxiety disorder, PTSD, and substance abuse), subset by Care Assessment Needs (CAN) Scores and across all time periods. Area Deprivation Index (ADI) is a measure of neighborhood disadvantage. Future Exposure Zone (FEZ) describes where each veteran resides but uses future hurricane exposure to determine which exposure category they fall under. Race/ethnicity data was ascertained from Veterans Health Association electronic health records and are self-reported data.

[a]These values represent how much greater the post-hurricane hazard ratio is compared to the pre-hurricane hazard ratio.

[b]AI/NA = American-Indian/Native Alaskan, NHPI = Native Hawaiian and Other Pacific Islander

© 2025 Yip CS et al. *JAMA Network Open.*

eFigure 4. Hurricane Sandy, Depression

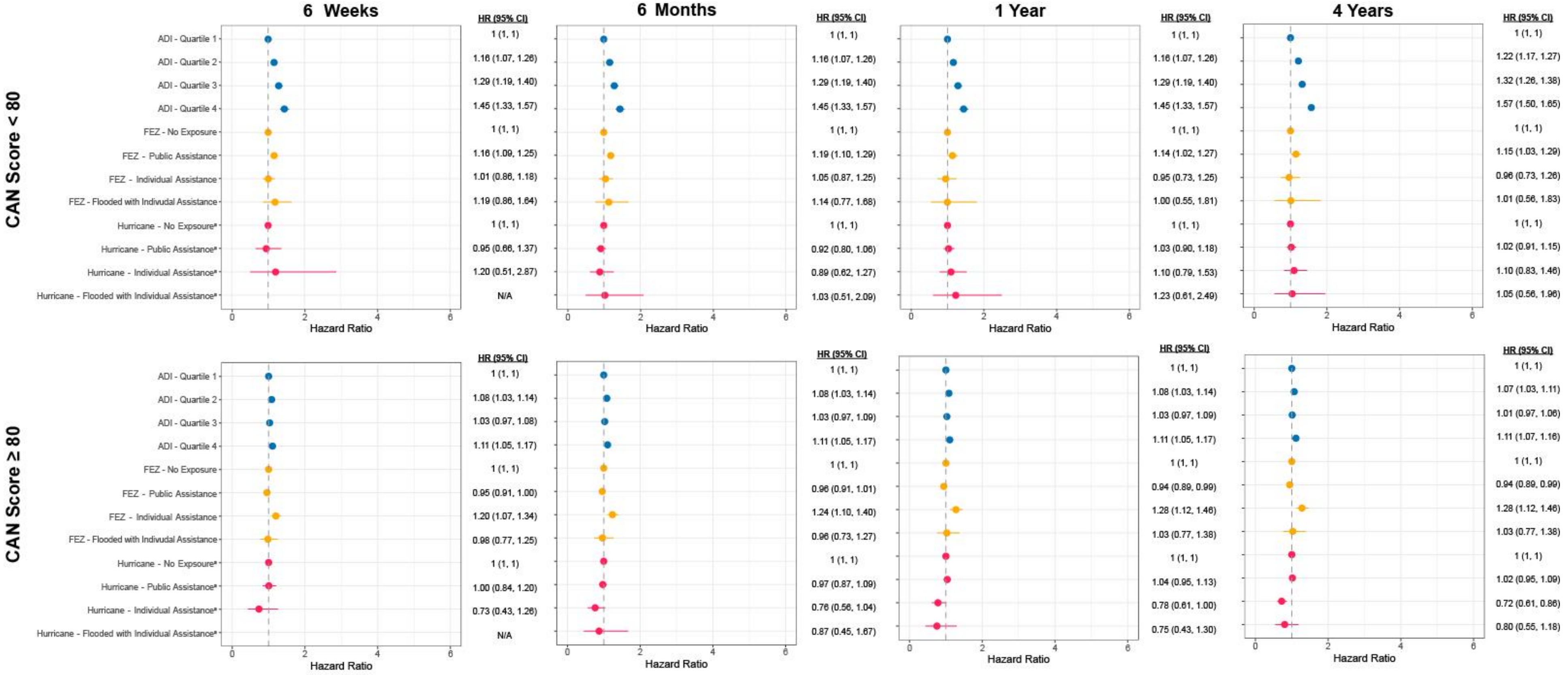

eFigure 4. Hazard ratio and 95% confidence intervals for the relationship between ADI, FEZ, and exposure to Hurricane Sandy, and UC/ED visits and in-patient hospitalizations for depression, subset by Care Assessment Needs (CAN) Scores and across all time periods.

[a]These values represent how much greater the post-hurricane hazard ratio is compared to the pre-hurricane hazard ratio.

© 2025 Yip CS et al. *JAMA Network Open.*

eFigure 5. Hurricane Harvey, Depression

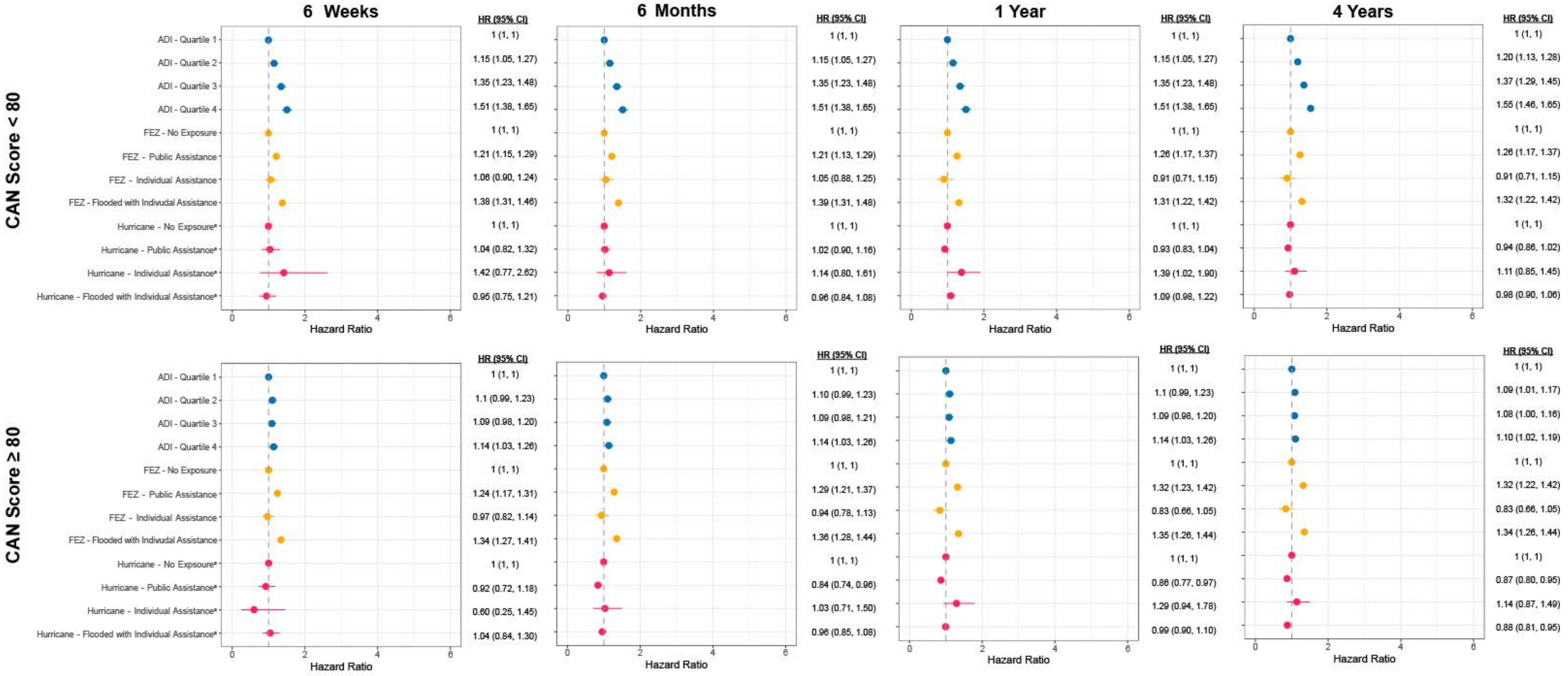

eFigure 5. Hazard ratio and 95% confidence intervals for the relationship between ADI, FEZ, and exposure to Hurricane Harvey, and UC/ED visits and in-patient hospitalizations for depression, subset by Care Assessment Needs (CAN) Scores and across all time periods.

[a]These values represent how much greater the post-hurricane hazard ratio is compared to the pre-hurricane hazard ratio.

© 2025 Yip CS et al. *JAMA Network Open.*

eFigure 6. Hurricane Sandy, Post-Traumatic Stress Disorder (PTSD)

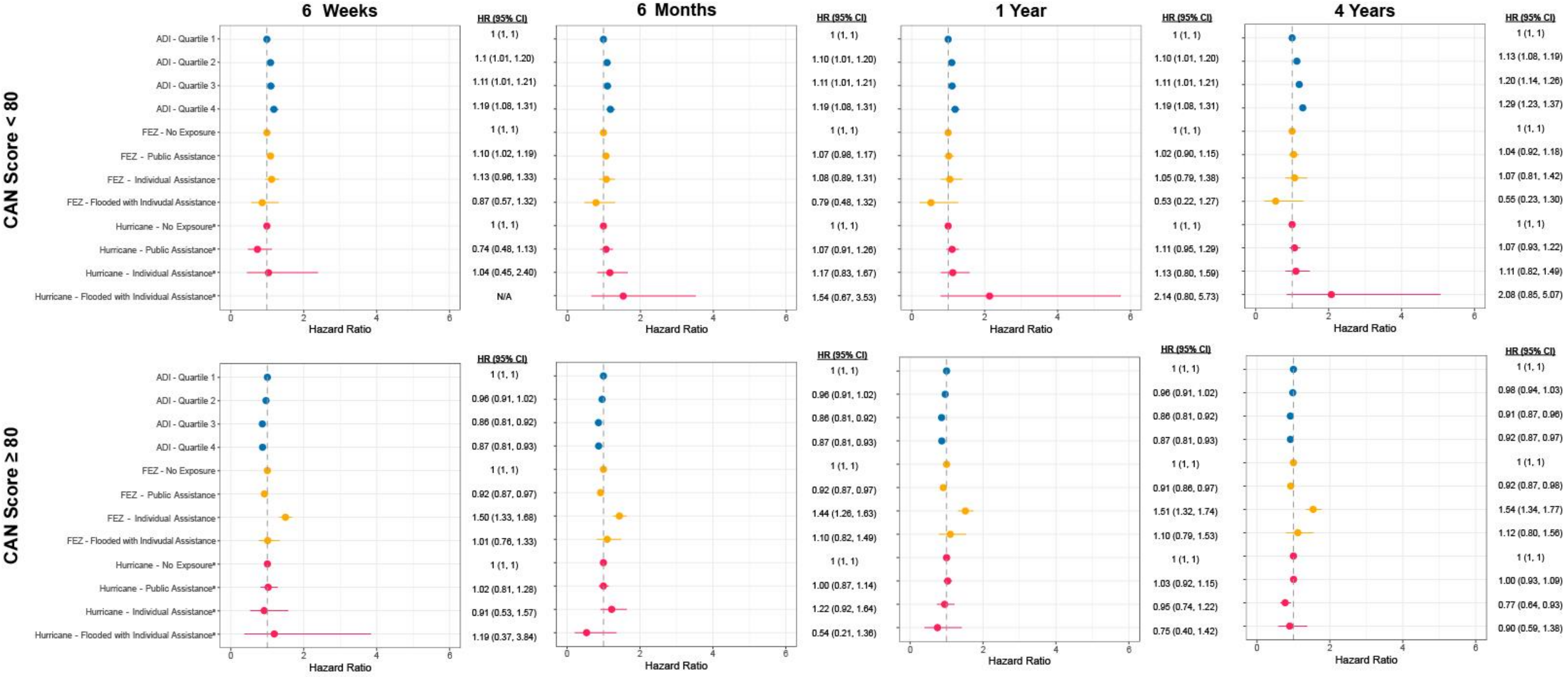

eFigure 6. Hazard ratio and 95% confidence intervals for the relationship between ADI, FEZ, and exposure to Hurricane Sandy, and UC/ED visits and in-patient hospitalizations for PTSD, subset by Care Assessment Needs (CAN) Scores and across all time periods.

[a]These values represent how much greater the post-hurricane hazard ratio is compared to the pre-hurricane hazard ratio.

© 2025 Yip CS et al. *JAMA Network Open.*

eFigure 7. Hurricane Harvey, Post-Traumatic Stress Disorder (PTSD)

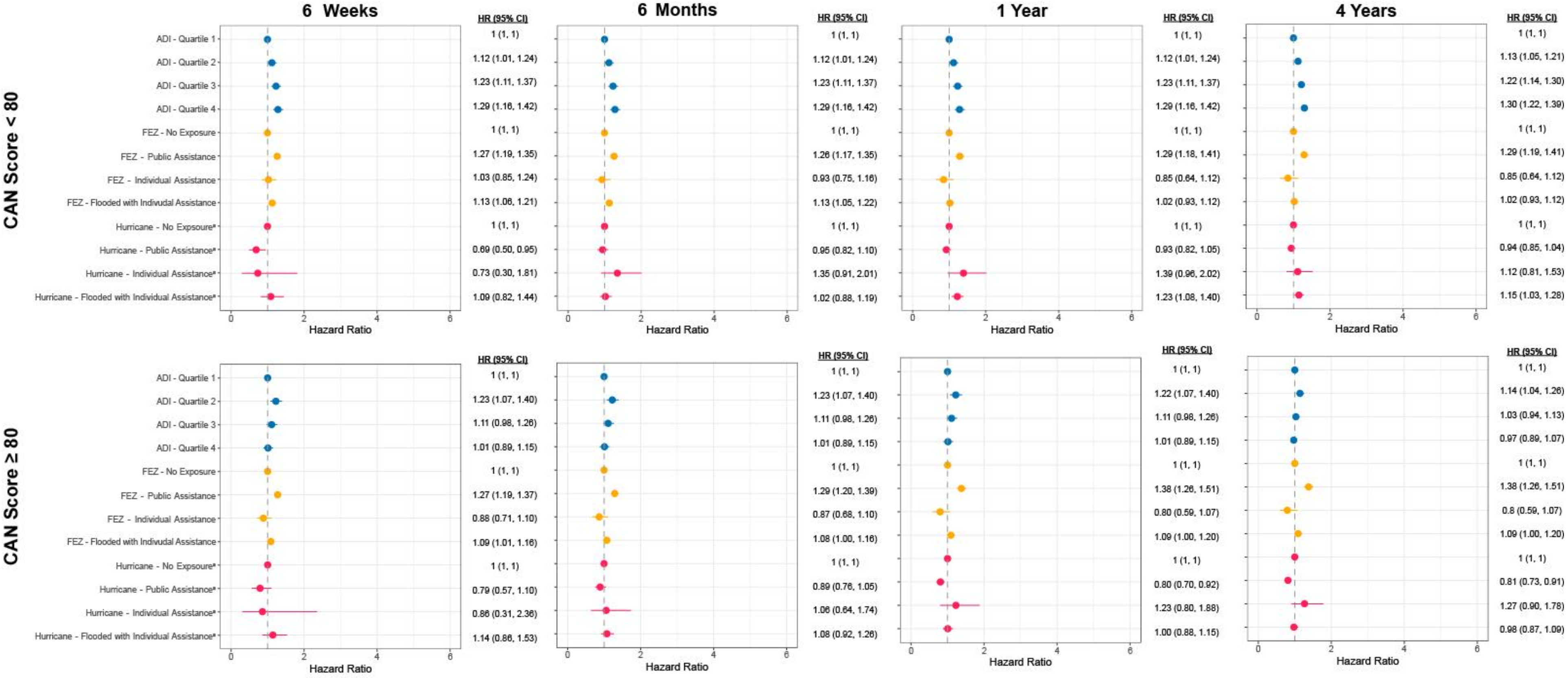

eFigure 7. Hazard ratio and 95% confidence intervals for the relationship between ADI, FEZ, and exposure to Hurricane Harvey, and UC/ED visits and in-patient hospitalizations for PTSD, subset by Care Assessment Needs (CAN) Scores and across all time periods.

[a]These values represent how much greater the post-hurricane hazard ratio is compared to the pre-hurricane hazard ratio.

© 2025 Yip CS et al. *JAMA Network Open.*

eFigure 8. Hurricane Sandy, Substance Abuse Disorder

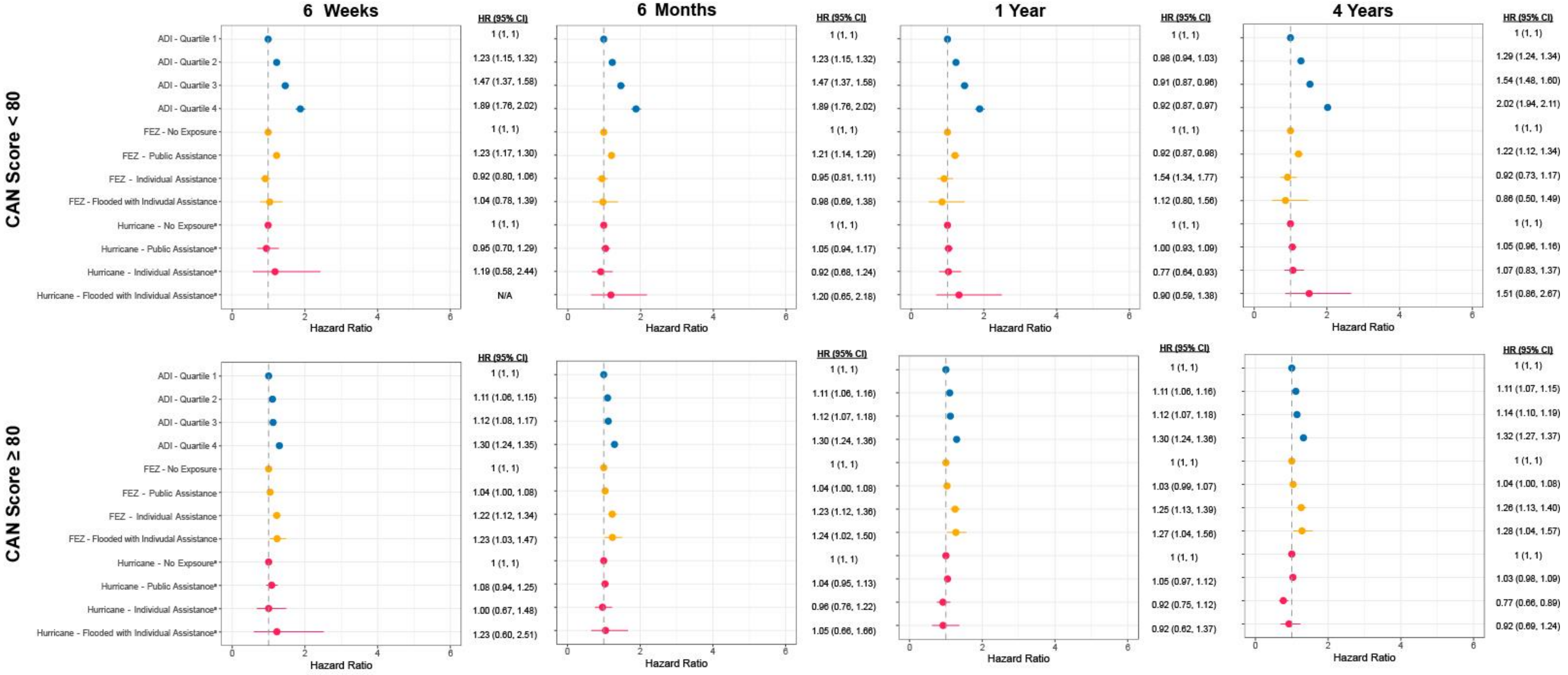

eFigure 8. Hazard ratio and 95% confidence intervals for the relationship between ADI, FEZ, and exposure to Hurricane Sandy, and UC/ED visits and in-patient hospitalizations for substance abuse disorder, subset by Care Assessment Needs (CAN) Scores and across all time periods.

[a]These values represent how much greater the post-hurricane hazard ratio is compared to the pre-hurricane hazard ratio.

© 2025 Yip CS et al. *JAMA Network Open.*

eFigure 9. Hurricane Harvey, Substance Abuse Disorder

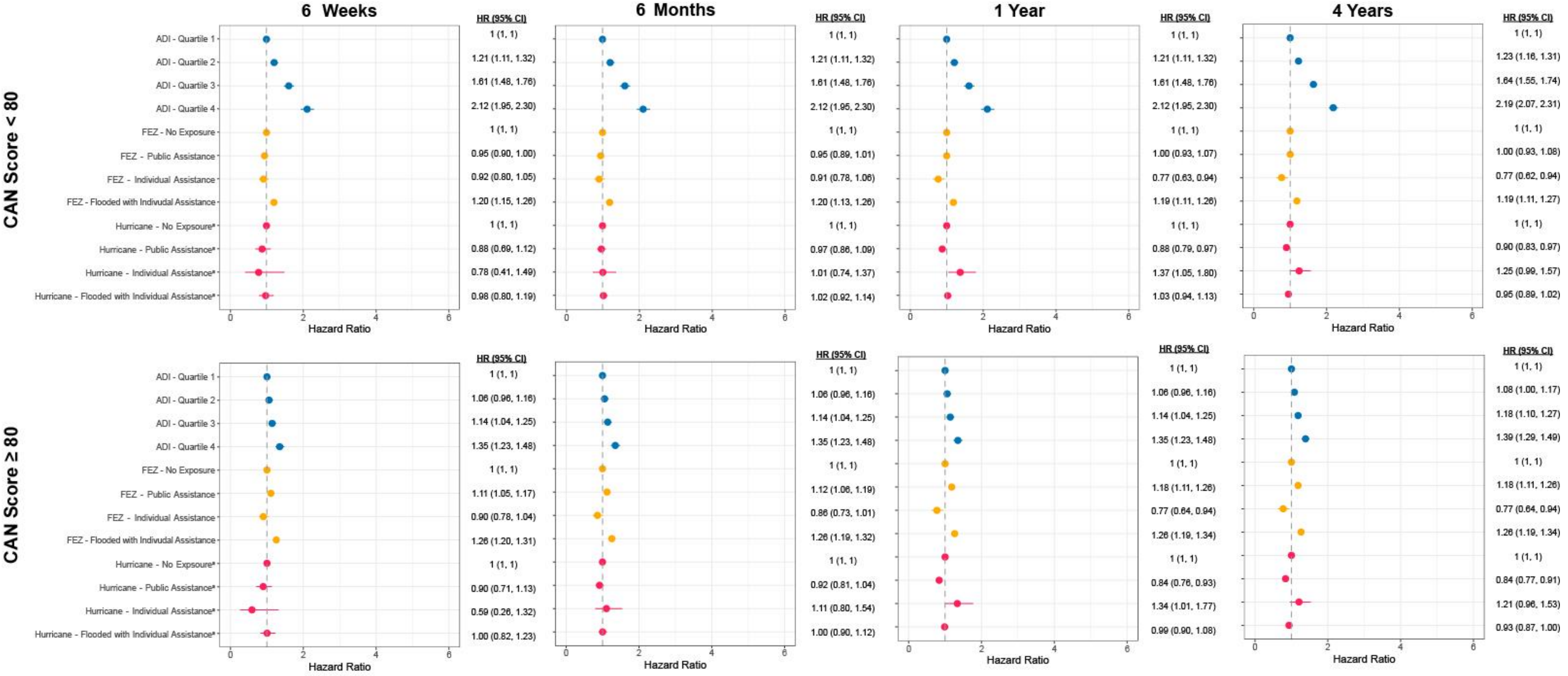

eFigure 9. Hazard ratio and 95% confidence intervals for the relationship between ADI, FEZ, and exposure to Hurricane Harvey, and UC/ED visits and in-patient hospitalizations for substance abuse disorder, subset by Care Assessment Needs (CAN) Scores and across all time periods.

[a]These values represent how much greater the post-hurricane hazard ratio is compared to the pre-hurricane hazard ratio.

© 2025 Yip CS et al. *JAMA Network Open.*
