# Supplementary material for: Neighborhood Disadvantage and the Association of Hurricanes Sandy and Harvey With Veterans’ Mental Health
Source: JAMA Netw Open. 2025 Jan 17;8(1):e2455013. doi: 10.1001/jamanetworkopen.2024.55013 (PMC11742530; doi:10.1001/jamanetworkopen.2024.55013)
Supplement: Supplement 2. — Data Sharing Statement [file jamanetwopen-e2455013-s002.pdf]

Data Sharing Statement

Yip. Neighborhood Disadvantage and the Association of Hurricanes Sandy and Harvey With Veterans' Mental Health. *JAMA Netw Open*. Published January 17, 2025. doi:10.1001/jamanetworkopen.2024.55013

Data
Data available: No

Additional Information
Explanation for why data not available: The data are available through the Veterans Health Administration:https://www.research.va.gov/programs/vinci/
